# The Evolution of Targeted Therapies in Early Hormone Receptor-Positive, HER2-Negative Breast Cancer

**DOI:** 10.3390/cancers18071130

**Published:** 2026-04-01

**Authors:** Hyejee Ohm, Caroline Lohrisch, Nathalie LeVasseur, Christine Simmons, Zahi I. Mitri

**Affiliations:** 1 Division of Medical Oncology, University of British Columbia, Vancouver, BC V5Z 4E6, Canada; hyejee.ohm@bccancer.bc.ca (H.O.);; 2 Medical Oncology, British Columbia Cancer Agency, Vancouver, BC V5Z 4E6, Canada

**Keywords:** breast cancer, targeted therapy, endocrine therapy, adjuvant CDK 4/6 inhibitors, genomic assays, bone-modifying agents, personalized medicine

## Abstract

Breast cancer is the second most common malignancy worldwide, and over 70% of new diagnosis are of the hormone receptor-positive (HR+) and HER2-negative (HER2−) subtype. The cornerstone in management of early stage HR+ breast cancer has historically consisted of chemotherapy and endocrine therapies. Genomic assays in early stage HR+HER2− breast cancer has provided a prognostic tool for recurrence and a predictive tool to select patients for adjuvant chemotherapy. The introduction of bone-modifying agents and adjuvant CDK 4/6 inhibitors into early stage disease represents new therapeutic modalities aimed at reducing risk in high-risk HR+HER2− cancers. This manuscript aims to provide a comprehensive overview of the evidence behind endocrine therapy and recommended duration of treatment, genomic assays to guide chemotherapy delivery, data guiding use of bisphosphonate therapy to reduce bone recurrence, and indications for incorporating CDK 4/6 inhibitors. In addition, novel endocrine agents and targeted therapies under investigation are highlighted.

## Simple Summary

Hormone receptor-positive breast cancer is the most common breast cancer subtype and represents a major public health issue worldwide. Endocrine therapy remains the cornerstone of treatment for early stage hormone receptor-positive breast cancer. Advances in treatment modalities and genomic testing platforms provide incremental benefits as well as opportunities to personalize therapies in this disease. Weighing these interventions against potential impact on short- and long-term quality of life highlights the vital shared decision-making process required in this patient population. This review aims to highlight the current evidence underlying treatment selection and emerging therapies in early stage hormone receptor-positive breast cancer. By examining these multifaceted approaches, we hope to achieve a better understanding of how precision medicine and innovative therapies are reshaping the standard of care to significantly improve patient outcomes and long-term survival rates.

## 1. Introduction

Breast cancer is the second most common malignancy worldwide, with 2.3 million new cases reported in 2022 [1]. It is a heterogeneous disease, classified into three subtypes based on immunohistochemical expression: hormone receptor-positive (HR+), human epidermal growth factor 2 (HER2)-positive (HER2+), and triple negative breast cancer (TNBC) [2]. HR+HER2− disease accounts for 70% of breast cancer diagnoses, defined as estrogen receptor (ER) and progesterone receptor (PR) positivity in ≥1% of tumour nuclei [3,4].

The link between estrogen and HR+ breast cancer was first suggested by Dr. George Beatson, a Scottish surgeon, in the 1880s, who reported disease remission in three women with advanced breast cancer following bilateral oophorectomies [5]. Beatson’s work laid the foundation of antihormonal therapy or endocrine therapy (ET) in the management of breast cancer.

In the century following Beatson’s era, the management of breast cancer has advanced significantly. This review summarizes key evidence supporting the use of targeted systemic therapies, including ET, bisphosphonates, and cyclin-dependent kinase 4/6 inhibitors (CDK4/6i) in the treatment of early stage HR+ breast cancer.

## 2. Methods

We conducted a search of the literature using PubMed/MEDLINE, Scopus, and the Cochrane Library. Keywords used included, but were not limited to, “breast cancer”, “estrogen positive”, “hormone receptor positive”, “early stage breast cancer”, “genomic assays”, “bisphosphonates”, “endocrine therapy”, and “targeted therapy”. Peer-reviewed articles published in English were included. Select conference abstracts that were relevant to the topic were included, and no publication was available at the time of the review.

Endocrine Therapy. We included studies spanning 1988 to 2025, a timeline intended to provide an overview of the evolution of endocrine and targeted therapies for early stage HR+HER2− breast cancer since the initial Tamoxifen adjuvant trial (1988). We focused on randomized registration trials, and studies reporting solely on adjuvant chemotherapy were not included in this review. The trial data reported includes clinical trial population, sample size, primary endpoints, and key findings.

Genomic Tests for Adjuvant Treatment. We included studies relevant to genomic assays currently recommended per the National Comprehensive Cancer Network Guidelines version 2.2026. We focused on registration trials, with data including clinical trial population, sample size, primary endpoints and key findings, especially with regard to the predictive and prognostic impact of the assay.

Adjuvant Bisphosphonates. We included studies between 2012 and 2022 relevant to the use of anti-resorptive therapies in adjuvant breast cancer. We included randomized trials as well as meta-analyses relevant to therapeutic approvals in this setting. Trial data reported includes clinical trial population, sample size, primary endpoints and key findings, especially with regard to impacts on bone health and bone metastases.

CDK 4/6 Inhibitors. We included adjuvant randomized trials pertaining to the three currently available CDK4/6 inhibitors (palbociclib, ribociclib, and abemaciclib). The trial data reported includes clinical trial population, sample size, primary endpoints and key findings, especially with regard to toxicity and survival outcomes.

## 3. Endocrine Therapy

### 3.1. Tamoxifen

Tamoxifen is a selective estrogen receptor modulator (SERM) initially developed in the 1960s as a contraceptive pill [6]. Observations that in epithelial cells, tamoxifen binds to the ER as an antagonist, inhibiting cellular proliferation, raised the question of its role in treating hormone-responsive breast cancer. In other tissues such as the bone, endometrium, and heart, it acts as an ER agonist, promoting estrogenic effects [7].

Numerous studies have examined the benefit of adjuvant tamoxifen given for two, five, and more than five years (Table 1). The Nolvadex Adjuvant Trial in 1988 was among the first, showing a 36% relative reduction in breast cancer events and a 29% relative reduction in mortality with two years of adjuvant tamoxifen compared to no adjuvant therapy in mostly postmenopausal patients [8]. Event-free survival (EFS) curves separated within 12 months of treatment, and overall survival (OS) curves diverged after 24 months.

The National Surgical Adjuvant Breast and Bowel Project (NSABP) B-14 trial, initiated in the early 1990s, reported a 42% relative improvement in recurrence-free survival (RFS) and 20% relative benefit in overall survival (OS) after 15 years of follow-up for women treated with five years of tamoxifen versus no treatment [9,10,11,12]. The continued separation of survival curves between years 2 and 5 supported a sustained benefit from a five-year course of treatment. It is important that extending tamoxifen beyond five years of therapy was not associated with additional benefit in the NSABP-14 trial [12].

These findings were confirmed by a large and robust meta-analysis from the Early Breast Cancer Trialists’ Group (EBCTG) conducted in 2005, which included 194 randomized trials of adjuvant ET. Five years of tamoxifen reduced the relative risk of distant recurrence by 41% and the relative risk of death by 34%, irrespective of chemotherapy, age, or tumour characteristics, and was significantly more effective than only 1–2 years [13], establishing five years as the standard of care in early stage HR+ breast cancer. Tamoxifen was one of the first and continues to be among the most effective targeted treatments for cancer.

Tamoxifen is associated with menopause-mimicking side effects such as vasomotor symptoms, including hot flushes and night sweats, mood changes, fatigue, and insomnia. It can also cause vaginal dryness, dyspareunia, and decreased libido. Around 16% of patients report increased vaginal discharge. Serious adverse effects include an increased risk of thromboembolism (2%) and, in postmenopausal women, an increased risk of uterine cancer (1%) [11].

**Table 1:** Clinical trials for Tamoxifen.

Clinical Trial	Population	Treatment Regimens	Endpoints	Findings
Nolvadex Adjuvant Trial, 1988 [8]	1285 patients	10 mg tamoxifen PO BID for 2 years vs. placebo	EFS, OS	5-year EFS favoured tamoxifen vs. placebo (RR 0.64, 95% CI 0.53–0.77). 5-year OS favoured tamoxifen vs. placebo (RR 0.71, 95% CI 0.58–0.88).
NSABP B-14, 2004 [9,10,11,12]	2892 patients	10 mg tamoxifen PO BID for at least 5 years vs. placebo	RFS, OS	15-year RFS favoured tamoxifen vs. placebo (78% vs. 65%, HR 0.58, 95% CI 0.50–0.67, *p* < 0.0001). 15-year OS favoured tamoxifen vs. placebo (71% vs. 65%, HR 0.80, 95% CI 0.71–0.91, *p* = 0.0008).
EBCTCG, 2015 [13]	51,000 patients	Tamoxifen for 5 years (group 1) vs. tamoxifen for 1–2 years (group 2) vs. placebo (group 3)	Recurrence, breast cancer mortality, death without recurrence, all-cause mortality	Allocation to 5 years of tamoxifen reduces the annual breast cancer death rate by 31%. 5 years is significantly more effective in reducing recurrence and breast cancer mortality than 1–2 years of tamoxifen (2*p* < 0.00001 for recurrence, 2*p* = 0.01 for breast cancer mortality).

### 3.2. Aromatase Inhibitors

Aromatase is an enzyme that converts androgens to estradiol. In postmenopausal women, peripheral tissues such as the adrenal glands, skin, muscle, and adipose tissue become the primary source of estrogen production via the activity of aromatase. Aromatase inhibitors (AIs) block this enzyme, suppressing estrogen levels [14]. Following demonstration of efficacy in metastatic HR+ breast cancer, their role in adjuvant therapy was explored (Table 2).

The Arimidex, Tamoxifen, Alone or in Combination (ATAC) trial published in 2002 evaluated the non-steroidal AI (NSAI) anastrozole in postmenopausal women with early stage, node-negative or node-positive, HR+ breast cancer. Randomization was to anastrozole alone, tamoxifen alone, or anastrozole and tamoxifen combination for five years of treatment [15,16,17]. At 10-year follow-up, anastrozole achieved a 14% relative improvement in disease-free survival (DFS) compared to tamoxifen in HR+ patients, whereas the combination arm was no better than tamoxifen alone. There was no significant difference in OS between the treatment groups at 10 years of follow-up [15,17].

Letrozole, an NSAI, also showed improved efficacy over tamoxifen in the Breast International Group (BIG) 1–98 trial, which reported a 9% relative improvement in 10-year DFS with five years of therapy compared to tamoxifen [18,19,20,21]. Letrozole and anastrozole were proven to have comparable efficacy rates in the Femara versus Anastrozole Clinical Evaluation (FACE) trial [22].

A 2015 meta-analysis by the EBCTCG compared five years of AI therapy to tamoxifen of the same duration. The study included individual data sets from a total of nine trials on 35,718 women. AI-based therapy was associated with a 30% relative reduction in recurrence and a 15% relative reduction in breast cancer mortality compared to tamoxifen [23]. These results established AIs as a part of the standard of care of adjuvant treatment for postmenopausal women with early stage HR+ breast cancer.

AIs are associated with similar menopausal-type side effects as well as genitourinary and sexual dysfunction symptoms as tamoxifen [24]. AIs also increase cardiovascular risk through effects such as dyslipidemia; however, the observed rise in cholesterol is relative to tamoxifen, which is known to lower cholesterol, rather than to the patient’s baseline [16,25]. AI therapy contributes to bone loss and is associated with a higher risk of fractures (8% with AIs vs. 5% with tamoxifen) [23]. Unlike tamoxifen, AIs are not associated with an increased risk of thromboembolism or uterine cancer [23].

It is important to note that these registration trials for AI therapy enrolled only post-menopausal women and did not include contemporary genomic assays for enhanced prognostic data on risk of recurrence, especially distant metastatic recurrence. Further, in the absence of OS benefit for AI compared to Tamoxifen, decisions for adjuvant therapy should take into consideration patient factors, co-morbidities, as well as adherence to a prolonged treatment to achieve the intended breast cancer risk reduction benefit.

**Table 2:** Clinical trials for aromatase inhibitors.

Clinical Trial	Population	Treatment Regimens	Endpoints	Findings
ATAC, 2010 [16,17]	48,473 patients	Tamoxifen 20 mg PO daily for 5 years vs. anastrozole 1 mg PO daily for 5 years vs. anastrozole and tamoxifen combination therapy	DFS, time to recurrence, time to distant recurrence, contralateral breast cancer, death after recurrence, and OS	Combination group was discontinued after initial analysis, as there was no efficacy or tolerability benefit. The 10-year follow-up showed DFS (HR 0.91, 95% CI 0.83–0.99, *p* = 0.04), time to recurrence (HR 0.84, 95% CI 0.75–0.93, *p* = 0.001), and time to distant recurrence (0.87, 95% CI 0.77–0.99, *p* = 0.03) in favour of anastrozole.
BIG 1–98, 2011 [18,19,20,21]	8010 patients	Letrozole 2.5 mg PO daily for 5 years vs. tamoxifen 20 mg PO daily for 5 years vs. letrozole for 2 years followed by tamoxifen for 3 years vs. tamoxifen for 2 years followed by letrozole for 3 years	DFS, OS, distant recurrence-free interval, invasive BCFI, breast cancer mortality	8-year DFS (HR 0.82, 95% CI 0.74–0.92, *p* = 0.0002) and OS (HR 0.79, 95% CI 0.69–0.90, *p* = 0.0006) favoured letrozole over tamoxifen.
FACE, 2017 [22]	4136 patients	Letrozole 2.5 mg PO daily for 5 years or anastrozole 1 mg PO daily for 5 years	DFS, OS, safety	5-year DFS was 84.9% for letrozole vs. 82.9% for anastrozole (HR 0.93, 95% CI 0.80–1.07, *p* = 0.3150). 5-year OS is similarly not significantly different.
EBCTCG, 2015 [23]	35,129 patients	AI for 5 years (group 1) vs. tamoxifen for 5 years (group 2) vs. tamoxifen for 2–3 years followed by an AI to year 5 (group 3) vs. AI for 2–3 years followed by tamoxifen to year 5 (group 4)	Recurrence, breast cancer mortality, death without recurrence, and all-cause mortality	AIs reduce recurrence rates by about 30% compared with tamoxifen, while treatments differ but not thereafter. 5 years of AI reduces 10-year breast cancer mortality by 15% compared with 5 years of tamoxifen.

### 3.3. Switching Treatment

Several clinical trials have investigated the outcomes of switching ET in perimenopausal and postmenopausal women (Table 3). The ABCSG-8/ARNO-95 trial in 2005 compared five years of tamoxifen to two years of tamoxifen followed by an additional three years of AI. Patients who switched to an AI had improved two-year EFS (96% vs. 93%) [26]. The results of the Intergroup Exemestane Study (IES), which evaluated 5 years of tamoxifen to 2–3 years of tamoxifen followed by exemestane for a total of 5 years, were concordant [27].

The BIG 1–98 trial in 2011 also evaluated ET sequencing. In addition to comparing letrozole and tamoxifen monotherapy, it included two sequencing arms: letrozole for two years followed by tamoxifen for three years, and tamoxifen for two years followed by letrozole for three years. Neither sequencing strategy demonstrated differences in DFS or OS compared to letrozole monotherapy [19]. This observation is corroborated by the EBCTCG meta-analysis in 2015, which found that DFS and breast cancer mortality were affected only when treatments differed, with outcomes between sequencing and AI monotherapy groups converging when patients were switched from tamoxifen to an AI for at least two years of the planned five-year course [23].

Since the tolerability of AIs and tamoxifen is a major determinant of adherence to five years of therapy, and side-effect profiles differ for individual patients, switching therapy can be a strategy to improve adherence and quality of life. This approach includes switching between different AI agents to help maintain patients on therapy, or between Tamoxifen and AI after 2–3 years for risk reduction. Importantly, a switch from Tamoxifen to AI should include a shared decision-making process with patients, outlining potential benefits, risks, as well as additional monitoring and potential interventions such as anti-resorptive therapy to mitigate the risk of fractures associated with AI therapy.

### 3.4. Extended Therapy

The benefits noted with five years over shorter durations prompted further investigation into the optimal duration of ET (Table 4). Fifteen-year pooled analysis of the Adjuvant Tamoxifen: Longer Against Shorter (ATLAS) and the Adjuvant Tamoxifen—To Offer More (aTTOM) trials, which compared 10 to 5 years of adjuvant tamoxifen, demonstrated a 25% relative improvement in RFS and 28% relative improvement in OS with 10 years of treatment. In a follow-up to the ATLAS trial, the differences between the two groups increased over time, with an absolute difference in RFS of 3.7% at 15 years from 1.4% at 10 years [28,29]. While these results differ from the NSABP-14 trial, which reported no benefit from extended Tamoxifen beyond five years, it is important to note that NSABP-14 included node-negative patients only, while the ATLAS study did include approximately 40% of participants with node-positive breast cancer.

The MA.17 trial and the NSABP B-42 trial explored whether an additional five years with an AI would be beneficial. In MA.17, patients who had received 4.5–6 years of prior tamoxifen were randomized to an additional 5 years of letrozole or placebo. Patients who received treatment for 10 years had an improved five-year DFS (95% vs. 91%, HR 0.66, *p* = 0.01), corresponding to a 4% absolute or 34% relative risk reduction, though OS was similar [30]. The NSABP B-42 trial, which randomized patients after five years of tamoxifen or AI to another five years of letrozole or placebo, showed a trend toward improved DFS at seven years (84.7% vs. 81.3%, HR 0.85, *p* = 0.048). While the difference in outcomes was not statistically significant in NSABP-42, the absolute risk reduction (3.4%) was similar to that of MA-17 [31].

**Table 4:** Clinical trials for extended therapy.

Clinical Trial	Population	Treatment Regimens	Endpoints	Findings
ATLAS, 2013 [28]	12,894 patients	After 5 years tamoxifen, patients randomized to stop tamoxifen or continue to year 10	Recurrence, side effects, breast cancer mortality, and overall mortality	Continued tamoxifen reduced risk of recurrence (21.4% with continued treatment vs. 25.1% with control, *p* = 0.002) and breast cancer mortality (12.2% with continued treatment vs. 15.0% with control, *p* = 0.01).
aTTom, 2013 [29]	6953 patients	After 5 years tamoxifen, patients randomized to stop tamoxifen or continue to year 10	Recurrence, mortality, and hospital admissions	Continued tamoxifen reduced risk of recurrence (17% with continued treatment vs. 19% with control, *p* = 0.0003) from year 7 onward.
MA.17, 2016 [30]	1918 patients	After 4.5–6 years of tamoxifen an additional 5 years of letrozole 2.5 mg PO daily vs. placebo	DFS, recurrence, OS, incidence of contralateral breast cancer, quality of life, and long-term safety	5-year DFS was 95% with letrozole and 91% with placebo (HR 0.66, 95% CI 0.48–0.91, *p* = 0.01). No differences in OS.
IDEAL, 2018 [32]	1824 patients	After 5 years of hormone therapy, an additional 5 years vs. 2.5 years of letrozole 2.5 mg PO daily	DFS, OS, distant DFS, and contralateral breast cancer	At 6.6 years, no significant difference in DFS (HR 0.92, 95% CI 0.74–1.16, *p* = 0.49), OS, and distant DFS. Reduction in contralateral breast cancer with 5 years of treatment (HR 0.39, 95% CI 0.19–0.81, *p* = 0.01).
NSABP B-42, 2019 [31]	3966 patients	After 5 years of AI or tamoxifen, an additional 5 years of letrozole 2.5 mg PO daily vs. placebo	DFS, second primary cancer, death, OS, and breast cancer-free interval	7-year DFS showed statistically non-significant difference of 81% vs. 85% (HR 0.85, 95% CI 0.73–0.999, *p* = 0.048).
ABCSG-16, 2021 [33]	3484 patients	After 5 years of hormone therapy, an additional 5 years vs. 2 years of anastrozole 1 mg PO daily	RFS, OS, contralateral breast cancer, second primary cancer, and clinical bone fracture	8-year DFS not different between 5-year vs. 2-year group (HR 0.99, 95% CI 0.85–1.15, *p* = 0.9). Clinical bone fracture risk is higher in the 5-year group than the 2-year group (HR 1.35, 95% CI 1.00–1.84).
DATA, 2023 [34]	1912 patients	After 2–3 years of tamoxifen, an additional 6 years vs. 3 years of anastrozole 1 mg PO daily	Adapted DFS (DFS beyond 3 years after randomization)	10-year adapted DFS was 69.2% in the 6-year group and 66% in the 3-year group (HR 0.86, 95% CI 0.72–1.01, *p* = 0.073). The absolute benefit of extended therapy increased when additional high-risk factors (lymph node-positive disease, tumours > 5 cm) were present.

The impact of endocrine therapy beyond 5 years, but less than 10 years, was also investigated, ideally to achieve maximum benefit from endocrine therapy while mitigating cumulative risks of extended treatment. The Optimal Duration of Extended Adjuvant Endocrine for Early Breast Cancer (IDEAL), ABCSG-16, and Extended Adjuvant Aromatase Inhibition after Sequential Endocrine Therapy (DATA) trials compared 10 years to 6–7.5 years of adjuvant ET. Most patients had N0 or N1 disease (80–90%), while a small proportion had N2/N3 involvement. All three trials found no statistically significant differences in DFS benefit with the extension to 10 years compared to 6–7.5 of ET [32,33,34]. In an exploratory subgroup analysis, however, the DATA trial found that in patients with high-risk factors such as lymph node involvement or primary tumours larger than 5 cm, 10 years of treatment translated into greater absolute benefits in DFS. For example, in patients with both lymph node involvement and primary tumours above 5 cm, the absolute DFS benefit of nine years compared to six years was 13.2%, compared to 3.2% in the overall cohort [34].

A 2025 meta-analysis by the EBCTCG of 12 RCTs examining extended adjuvant endocrine therapy in postmenopausal women found that after some exposure to AI and tamoxifen, extending adjuvant endocrine treatment for a median of eight years of treatment reduced recurrence by 3.6% and distant recurrence by 2%. The absolute benefit observed was greater for patients with node-positive disease at 3.8% compared to patients with node-negative disease at 2.7% [35].

In summary, for patients with N0 or N1 disease, the maximum DFS benefit seems to be reached with 7–8 years of adjuvant ET. It is important to note that these studies, including the EBCTG meta-analysis, focused on postmenopausal women. For N0/N1 disease, decisions on escalation of therapy for this population have to be taken into consideration, with novel prognostic genomic assays to balance potential risk compared to the benefit of extended therapies. Further, the type of initial endocrine therapy, whether tamoxifen or AI therapy, may impact the decision on extending therapy to provide patients exposure to AI at any point during the course to provide benefit for breast cancer recurrence.

In patients with higher risk disease, such as N2/N3 disease or large primary tumours, up to 10 years of ET can be considered if tolerated by the patient.

### 3.5. Ovarian Function Suppression

Premenopausal women are under-represented in early adjuvant ET trials, although meta-analyses show equal benefit of tamoxifen regardless of age and menopausal status. AIs cannot be used in premenopausal women without concurrent ovarian function suppression (OFS) or ovarian ablation [36]. However, AIs have been studied in combination with OFS, achieved either with gonadotropin-releasing hormone (GnRH) agonist injections, which have the advantage of being reversible, or through surgical oophorectomy.

The SOFT and TEXT trials evaluated whether the addition of OFS to ET was superior to tamoxifen alone. The SOFT trial randomized premenopausal women to tamoxifen alone, tamoxifen with OFS, or the steroidal AI exemestane with OFS for five years. At 15 years of follow-up, the absolute difference in DFS compared to tamoxifen alone was 3.5% with tamoxifen-OFS and 6.5% with exemestane-OFS [37,38,39,40].

The TEXT trial compared tamoxifen-OFS to exemestane-OFS for five years. A combined analysis of SOFT and TEXT studies with 15 years of follow-up demonstrated a DFS benefit of 3.6% with exemestane-OFS compared to tamoxifen-OFS [40].

No significant differences in OS were observed between treatment arms in the overall population at 15 years in either study. However, a 3–4% OS benefit was seen at 15 years for OFS-exemestane compared with OFS-tamoxifen among patients who received chemotherapy. Additionally, patients < 35 years of age had a 14% improvement in 15-year OS with exemestane-OFS and 10% improvement with tamoxifen-OFS compared to tamoxifen, and patients with grade 3 tumours had a 15-year OS benefit of 8% with exemestane-OFS [40].

Adherence to ET is limited by side effects, and toxicity increases with treatment intensification. In SOFT and TEXT, grade ≥ 3 adverse events occurred in 25% of patients on tamoxifen alone, 31% with tamoxifen-OFS, and 32% with exemestane-OFS. In a combined analysis, approximately 20–25% of participants discontinued protocol-assigned therapy earlier, reflecting concern for adherence to prolonged regimens and the impact of these treatments on patient quality of life. Osteoporosis is more common with exemestane-OFS (15%) compared to tamoxifen-OFS (7%) or tamoxifen (4%). Alterations in sexual functioning (vaginal dryness and dyspareunia), urogenital atrophy (increased urinary tract infections and vaginal stenosis) and metabolic abnormalities (hyperglycemia) were all higher in the OFS group [37]. These findings highlight the trade-offs of treatment escalation, especially in younger patients.

Results from SOFT and TEXT demonstrate that adding OFS to adjuvant ET improves DFS in premenopausal women, particularly those with high-risk disease, young age, or who received chemotherapy. While the addition of OFS offers greater efficacy than single-agent ET, potential side effects and tolerability must be carefully weighed in treatment decisions. Notably, the SOFT and TEXT trials planned for five years of therapy, and do not provide insights into the benefits of extended therapy in this patient population. This highlights a gap in our current treatment approach for premenopausal women beyond the initial five years of endocrine therapy, where no randomized trials are currently available. A careful inspection of risk of recurrence, tolerance to treatment, including risk of cumulative side effects, and co-morbidities can help navigate the complex and data-free zone of extended therapy beyond five years in this population.

## 4. Genomic Tests for Adjuvant Treatment

Genomic assays are now commonly used to improve prognostic estimates in HR+HER2− early stage breast cancer, and to guide decisions about the value of chemotherapy in reducing disease recurrence.

### 4.1. Oncotype Dx

Oncotype Dx (Exact Sciences, Madison, WI, USA) is a 21-gene assay performed on tumour tissue, generating a risk score (RS) ranging from 0 to 100. Oncotype was initially shown to be prognostic, with higher RS associated with a higher risk of recurrence. The assay was retrospectively developed and validated in early stage HR+ breast cancer patients treated with tamoxifen with or without chemotherapy in the NSABP B14 and B20 trials. The test for interaction between RS and chemotherapy treatment was statistically significant. Patients with high risk (RS ≥ 31) experienced an absolute reduction in distant recurrence risk of 28% with chemotherapy, in contrast to only a 1% benefit from chemotherapy in patients with low risk scores (RS < 18) [41].

TailorX was a prospective study that randomized patients with early stage node-negative HR+ breast cancer planned for adjuvant ET and an intermediate oncotype RS to chemotherapy or no chemotherapy. The study used a more conservative estimate of intermediate risk, RS 11–25, to minimize the chance of undertreating patients. Overall, in the intermediate-risk group, there were no differences in nine-year DFS or OS between patients who did and did not receive chemotherapy. However, in patients younger than 50 years of age, chemotherapy was associated with a reduction in distant recurrence of 1.6% for RS 16–20 and of 6.5% when RS was 21–25 [42]. In women 50 and older, there was no benefit for chemotherapy for the range of RS between 11 and 25.

The RxPonder trial randomized patients with early stage HR+ breast cancer, 1–3 positive axillary lymph nodes, and RS ≤ 25 to chemotherapy or no chemotherapy. In postmenopausal patients, five-year invasive DFS was 91% with chemotherapy and 92% without chemotherapy. In premenopausal women, however, the five-year invasive DFS was significantly higher at 94% with chemotherapy compared to 89% without chemotherapy [43]. It is unclear how much of the chemotherapy benefit comes from the induction of menopause in contrast to its cytotoxic effects, particularly in the lower end of the RS range. There are ongoing clinical trial efforts (e.g., OFSET trial NRG BR009) to better understand the role of chemotherapy in the premenopausal population [44].

In summary, the Oncotype DX assay is validated for use in both node-negative and node-positive (N1) postmenopausal patients, as well as in node-negative premenopausal women. As a prognostic assay, it provides information on the distant risk of recurrence. As a predictive assay, the RS score does provide estimates of the potential absolute benefit of chemotherapy. In early stage node-negative HR+ breast cancer patients and RS ≤ 25, adjuvant ET alone is recommended. Patients 50 and under may derive some benefit from chemotherapy when their RS is in the 21–25 range. In early stage HR+ breast cancer patients with 1–3 positive lymph nodes who are postmenopausal, chemotherapy can be avoided if RS ≤ 25; however, it should be considered in premenopausal women regardless of RS.

### 4.2. MammaPrint

MammaPrint (Agendia, Irvine, CA, USA) is another multigene assay (70 genes) performed on a tumour sample, and results in classification into four risk groups: ultra-low, low, high 1, and high 2. The MINDACT trial prospectively evaluated the added utility of MammaPrint over standard clinicopathologic criteria in predicting treatment benefit in early stage HR+ breast cancer. Patients with discordant clinical and genomic risk—defined as either low clinical risk with high genomic risk or high clinical risk with low genomic risk—were randomized to receive either adjuvant chemotherapy or ET alone. At 5 years, there was only a 1.5% difference in DFS for patients with high clinical and low genomic risk who did and did not receive adjuvant chemotherapy. In the MINDACT trial, incorporating MammaPrint reduced adjuvant chemotherapy use in 46% of patients. In patients with low clinical and high genomic risk, the absolute difference in DFS was <1% between those who did and did not receive chemotherapy [45].

With a longer follow-up of the MINDACT trial, a difference in chemotherapy benefit emerged for age. Patients over 50 years with discordant clinical and genomic risk were able to safely avoid chemotherapy, while younger patients saw a distant metastasis-free survival difference of 5%, favouring chemotherapy (94% vs. 89%) [46]. Notwithstanding cross-trial comparisons, this difference level mimics what was observed in the RxPonder trial with Oncotype Dx.

The MammaPrint assay provides prognostic information for patients with node-negative or node-positive early stage HR+ breast cancer. In a select population (age > 50) with high clinical risk, Mammaprint can help determine whether adjuvant chemotherapy may be safely omitted based on genomic risk stratification. Mammaprint, however, carries little predictive value for the benefit of chemo in patients with clinically low-risk disease. It is worth noting that further stratification of Mammaprint High Risk results into Mammaprint-High 1 and Mammaprint-High 2 is under evaluation to determine the benefit of anthracycline chemotherapy, as well as the potential benefit of immune checkpoint blockade in early stage HR+ breast cancer.

### 4.3. Prosigna

Similar to the previous two assays, Prosigna (NanoString Technologies, Seattle, WA, USA) measures the expression of 50 genes in breast tumour tissue, generating a risk of recurrence (ROR) score ranging from 0 to 100. For node-negative patients, ROR is categorized as low (0–40), intermediate (41–60), or high (61–100) [47,48].

Evidence supporting Prosigna comes from retrospective analyses of existing clinical trials. A secondary analysis of the ATAC trial assessed six prognostic assays in early stage HR+ breast cancer, including the Prosigna ROR. Among node-negative patients, the 10-year risk of recurrence was 3% for low, 14% for intermediate, and 32% for high ROR scores. In patients with 1–3 positive lymph nodes, only 8% had a low ROR, suggesting that in over 90% of cases, ROR would not alter treatment recommendations in this group [49].

The ROR score is associated with response to anthracycline-based adjuvant chemotherapy in early stage breast cancer. The DBCG89D trial randomized patients with high-risk disease, defined as: (1) premenopausal, node-negative, grade 2–3 tumours; (2) premenopausal, node-positive, HR+ tumours; or (3) postmenopausal, node-negative, HR– tumours. Patients were randomized to CMF (cyclophosphamide, methotrexate, fluorouracil) or CEF (cyclophosphamide, epirubicin, and fluorouracil) chemotherapy regimens. The hazard ratios for CEF versus CMF efficacy were 1.01 (95% CI, 0.59–1.73), 0.78 (95% CI, 0.53–1.15), and 0.54 (95% CI, 0.36–0.80) in patients with low, intermediate, and high ROR scores, respectively [50].

Unlike MammaPrint and Oncotype DX, Prosigna lacks validation from a prospective clinical trial. Nevertheless, the Prosigna assay is approved in many jurisdictions for use in postmenopausal patients with node-negative early stage breast cancer. It is not yet recommended for use in premenopausal patients as it has not been tested sufficiently in this population.

### 4.4. Breast Cancer Index

Oncotype DX, MammaPrint, and Prosigna are the genomic assays most used in clinical practice to guide adjuvant chemotherapy decisions. In contrast, the Breast Cancer Index (BCI) (Biotheranostics, San Diego, CA, USA) is the only genomic assay validated to predict the benefit of extended endocrine therapy. BCI consists of two gene expression biomarkers—a molecular grade index that assesses tumour proliferation, and HOXV13/IL17BR (H/I ratio), which predicts response to endocrine therapy. BCI categorizes patients into low, intermediate, and high risk. The intermediate and high BCI groups have a five-year distant recurrence rate that is 2.6% and 18% higher compared to the BCI low group, respectively [51].

The Trans-aTTOM study performed BCI testing on patients previously enrolled in the aTTOM trial. Among node-positive patients, those with BCI (H/I)-high status derived significant benefit from extended tamoxifen therapy (10 vs. 5 years), with an absolute improvement in recurrence-free survival of 9.7%. In contrast, BCI (H/I)-low patients showed no benefit from extended therapy, with an absolute difference of −1.2% [52,53].

The BCI has the highest utility in postmenopausal women with node-positive early stage HR+ breast cancer and provides a predictive assay in that setting for benefit from extended adjuvant endocrine therapy. It is important to note that BCI is usually completed as patients are completing their initial five-year course of therapy, and patients may have had another genomic assay completed at initial diagnosis for prognostication as well as prediction of chemotherapy benefit. It is not clear to date whether the information from baseline genomic assay adds to the BCI predictive value for extended endocrine therapy.

### 4.5. Concordance of Genomic Tests

In the TransATAC study, Buus et al. evaluated Oncotype DX and Prosigna to assess concordance and to identify the molecular features underlying their differences. The analysis included early stage, hormone receptor-positive, postmenopausal patients from the ATAC trial who did not receive chemotherapy but completed at least five years of tamoxifen or an AI. Overall, Oncotype DX showed weaker correlations with Prosigna (ρ = 0.32) [54]. Molecular profiling indicated that this discordance was primarily due to Oncotype DX’s stronger weighting of estrogen-related genes, whereas Prosigna relied more heavily on proliferation-related features.

Discordance has also been observed between Oncotype Dx and MammaPrint. In a single-institution study of 437 patients, Dabbs et al. reported that among those classified as low risk by MammaPrint, only 63% had concordant Oncotype Dx results. Similarly, among patients categorized as intermediate risk by Oncotype Dx, concordance with MammaPrint was observed in only 65% of cases [55]. A separate study by Maroun et al. further confirmed a concordance rate of approximately 60–70% between the two assays [56].

While different genomic assays have been investigated in similar populations, it remains important for clinicians to consider clinical risk, patient factors, and patient preferences when deciding on a genomic assay. Further, it is important to understand the contribution of each assay, from a prognostic and predictive manner, to help decide and finalize systemic therapy recommendations. Further, there is to date no clear data to guide the use of multiple assays at initial diagnosis. Given the potential discordance reported in the literature, it remains preferred to perform one genomic assay for eligible patients at diagnosis to avoid confusion in final treatment decisions.

## 5. Adjuvant Bisphosphonates

Bone metastases are one of the most common sites of breast cancer metastases, occurring in approximately 70% of cases. Patients with bone metastasis are at risk for adverse skeletal events such as fractures, hypercalcemia, and spinal cord compression [57]. Additionally, bone resorption in the setting of bone metastases promotes tumour growth. Tumour cells release signalling factors that stimulate osteoblasts to produce the receptor activator of nuclear factor kappa beta ligand (RANKL). RANKL then activates osteoclasts via the RANK receptor, promoting osteolysis and the release of bone-derived growth factors, which further fuel tumour proliferation [58,59]. Based on this mechanism, it was hypothesized that bisphosphonates, agents that inhibit osteoclast function, might reduce the development of eventual bone metastases in early breast cancer (Table 5).

Many trials, including the NSABP B-34 trial with oral clodronate, the AZURE trial with zoledronic acid for five years, and the ABCSG-12 trial with zoledronic acid for three years, yielded negative results [60,61,62]. Notably, the AZURE trial ran exploratory subgroup analyses with patients stratified by menopausal status. While no DFS benefit was seen in premenopausal or perimenopausal patients, women more than five years into menopause experienced a 33% relative reduction in bone recurrence with zoledronic acid [61].

A 2015 EBCTCG meta-analysis of nearly 19,000 women confirmed a benefit of bisphosphonates in postmenopausal patients, with absolute reductions of 2.2% in bone recurrence and 3.3% in breast cancer mortality at 10 years [63]. A similar benefit was not seen for premenopausal women. Adjuvant bisphosphonate therapy is now recommended for all postmenopausal women with early breast cancer as per the American Society of Clinical Oncology (ASCO) guidelines [64].

Denosumab, a monoclonal antibody against RANKL, is an alternative to bisphosphonate therapy but is generally not recommended due to inconsistent results. The ABCSG-18 trial reported a 9-year DFS benefit of 3.5% with adjuvant denosumab compared to the placebo [65,66]. However, the D-CARE trial did not demonstrate any differences in bone metastasis-free survival [67]. Given the current discrepancy in results, the NCCN guidelines currently do not recommend the use of Denosumab in the adjuvant setting for early stage HR+ breast cancer.

**Table 5:** Clinical trials on adjuvant bisphosphonates.

Clinical Trial	Population	Treatment Regimens	Endpoints	Findings
NSABP B-34, 2012 [60]	3323 patients	Adjuvant systemic treatment alone vs. oral clodronate for 3 years	DFS, OS, RFS, bone metastasis-free interval, and non-bone metastasis-free interval	8-year DFS, OS, RFS, and bone metastasis-free interval were not different between groups.
AZURE, 2014 [61]	3360 patients	Adjuvant systemic treatment alone vs. addition of zoledronic acid for 5 years	DFS, invasive DFS, OS, time to bone metastases, time to distant recurrence	7-year DFS did not differ between groups (HR 0.94, 95% CI 0.82–1.06, *p* = 0.3). Zoledronic acid reduced the development of bone metastases.
ABCSG-12, 2015 [62]	1803 patients	Adjuvant systemic treatment alone vs. addition of zoledronic acid for 3 years	DFS, RFS, OS	8-year DFS favours zoledronic acid (88% with zoledronic acid vs. 85% with control, HR 0.77, 95% CI 060–0.99, *p* = 0.042). No significant differences in OS.
EBCTCG, 2015 [63]	18,766 patients	Up to 1 year (group 1) vs. 2–5 years of adjuvant bisphosphonate (group 2) vs. placebo (group 3)	Recurrence, distant recurrence, breast cancer mortality, all-cause mortality, death without recurrence, bone recurrence as first distant recurrence, other first distant recurrence, locoregional recurrence, contralateral new primary breast cancer, bone fractures	No treatment effect in premenopausal women. Among postmenopausal women, 2.2% reduction in bone recurrence and 3.3% in breast cancer mortality.
SUCCESS A, 2021 [68]	2987 patients	Adjuvant zoledronic acid for 5 years vs. 2 years	DFS, OS, distant DFS, incidence of skeletal-related adverse events	DFS, OS, and distant DFS did not differ significantly between 5 vs. 2 years of zoledronic acid.
ABCSG-18, 2022 [65,66]	3425 patients	Adjuvant denosumab every 6 months vs. placebo	First clinical fracture, DFS, bone metastasis-free survival, and OS	Clinical fracture rate 5% vs. 10% with denosumab (HR 0.50, 95% CI 0.39–0.65). 9-year DFS benefit 3.5% (HR 0.83, 95% CI 0.71–0.97) with denosumab compared to placebo.
D-CARE [67]	4509 patients	Adjuvant denosumab for 5 years vs. placebo	Bone metastasis-free interval	The primary endpoint of bone metastasis-free survival was not significantly different between the groups (median not reached in either group; HR 0·97, 95% CI 0.82–1.14).

## 6. CDK4/6 Inhibitors

Cyclin-dependent kinase 4/6 inhibitors (CDK 4/6i) emerged as an effective anticancer treatment in the advanced HR+ breast cancer setting in 2015. CDK 4/6 enzymes regulate the cell cycle by promoting progression from G1 to S phase, committing cells to DNA replication and division. Inhibition of CDK 4/6 halts this process, suppressing tumour proliferation [69].

The first adjuvant study of CDK 4/6i in the adjuvant setting for early stage HR+ breast cancer was with palbociclib (Table 6). The PALLAS trial enrolled patients with stage II or III disease and assessed the addition of two years of palbociclib to standard ET, and the PENELOPE-B trial evaluated adding one year of palbociclib to ET in patients with residual disease after neoadjuvant chemotherapy. Both trials failed to demonstrate improvements in invasive DFS with the addition of palbociclib compared to ET alone [70,71,72].

The first positive result in early stage HR+ breast cancer came from the monarchE trial, which included patients with N2/N3 disease or N1 disease with additional high-risk features (tumour size ≥ 5 cm, histologic grade 3, or central Ki-67 ≥ 20%) (Table 6). Monarch-E evaluated the addition of two years of the CDK 4/6i abemaciclib to adjuvant ET, including tamoxifen or AI therapy, to show an improvement in 2-year invasive DFS from 88.7% with ET alone to 92.2%. The difference in DFS grew to 4.8% at three years, 6% at four years, and 7.6% at five years. Distant metastasis and distant DFS endpoints were also improved with abemaciclib, and recent OS data suggests 1.8% absolute lower risk of death at seven years [73,74,75]. The Food and Drug Administration (FDA) and Health Canada approved abemaciclib for high-risk early HR+ breast cancer in 2023.

Abemaciclib is associated with significant gastrointestinal side effects, including diarrhea, nausea, vomiting, and abdominal pain. Cytopenias, including neutropenia, anemia, and lymphopenia, are common. It is also associated with a small (1.6%) increased risk of symptomatic pneumonitis. There is also an increased risk of thromboembolism, which is of particular concern when used in combination with adjuvant tamoxifen [76].

A third CDK 4/6i, ribociclib, was studied in the NATALEE trial, in which patients with stage II or III HR+ disease were randomized to standard ET alone or ET with ribociclib for three years (Table 6). At five-year follow-up, the addition of ribociclib improved invasive DFS by 4.5%. Reduced metastatic recurrences and improved distant DFS were seen. Subgroup analyses demonstrated significant benefit regardless of nodal status (N0 or N1) or stage (II or III) [77,78,79]. Notably, the risk of recurrence in a subgroup of stage II node-negative patients was similar to that of stage III patients, further supporting the use of ribociclib in the node-negative high-risk population. Based on these results, the FDA approved ribociclib for early HR+ breast cancer in 2024, closely followed by Health Canada approval in 2025.

Common adverse effects associated with ribociclib include neutropenia, arthralgias, and liver-related toxicities. Dosing in the adjuvant trial was lower at 400 mg daily than the 600 mg daily in the metastatic setting, to reduce rates of grade 3 or higher side effects. Notable side effects include grade 3 or higher liver transaminitis rates of 10–15%, QT-interval prolongation at a rate of 5%, and a small (<1%) risk of severe cutaneous reactions, including Stevens–Johnson syndrome, toxic epidermal necrolysis, and drug rash with eosinophilia and systemic symptoms (DRESS) [78].

The majority of patients in both monarchE (98%) and NATALEE (88%) received prior chemotherapy [76,77]. Therefore, the benefit of adjuvant CDK 4/6i in women who did not require adjuvant chemotherapy remains uncertain and is actively being studied in the OFSET NRG-BR009 trial. This indicates that adjuvant CDK 4/6i does not currently represent an alternative treatment to chemotherapy, with the reported benefit largely reported in patients who received prior chemotherapy. From an endocrine therapy partner, abemaciclib can be administered with either tamoxifen or AI, while ribociclib was only studied with AI therapy, including in premenopausal women who required ovarian suppression. While this provides more endocrine therapy options for abemaciclib, it has to be weighed against the additive risk of thromboembolic events when combining tamoxifen and abemaciclib.

Further, there is currently a data gap for the management of post-menopausal patients with N1 disease and low recurrence score based on the oncotype test, as the RxPONDER trial reported, while MONARCH-E and NATALEE trials were ongoing. In this patient population, we currently face a dilemma of low genomic risk based on the oncotype and eligibility to escalate endocrine therapy if eligible for NATALEE or MONARCH-E criteria. This represents another area where shared decision-making with the patient becomes critical, and ensuring not to cause more harm with the limited data available. For patients who opt to proceed with adjuvant CDK 4/6 inhibition in this setting, the threshold for dose reduction or discontinuation should be low to avoid short and potentially long-term impacts on patients’ quality of life.

**Table 6:** Clinical trials for CDK4/6 inhibitors.

Clinical Trial	Population	Treatment Regimens	Endpoints	Findings
PALLAS, 2021 [70,71]	5760 patients	Adjuvant ET alone vs. palbociclib for 2 years	Invasive DFS, distant RFS, locoregional RFS, OS, safety	4-year invasive DFS not different between groups (84% with palbociclib vs. 85% with control, HR 0.96, 95% CI 0.81–1.14, *p* = 0.65).
PENELOPE-B, 2021 [72]	1250 patients	Adjuvant ET alone vs. palbociclib + ET for 1 year	Invasive DFS, distant DFS, OS, locoregional relapse-free interval, safety	3-year invasive DFS not different between groups (81% with palbociclib vs. 78% with control, HR 0.93, 95% CI 0.74–1.17, *p* = 0.525).
monarchE, 2024 [73,74,76]	5637 patients	Adjuvant ET alone vs. abemaciclib for 2 years	Invasive DFS, distant relapse-free survival, OS, patient-reported outcomes, safety	At 5 years, the absolute difference in invasive DFS was 7.6% (84% with abemaciclib vs. 76% with control, HR 0.68, 95% CI 0.60–0.77, *p* < 0.0001). At 7 years, an OS benefit of 1.8% (86.8% with abemaciclib vs. 85% with control, HR 0.84, 95% CI 0.72–0.98, *p* = 0.027).
NATALEE, 2025 [78]	7996 patients	Adjuvant ET alone vs. ribociclib for 3 years	Invasive DFS, distant DFS, RFS, OS, safety, quality of life	At 5 years, the absolute difference in invasive DFS was 4.5% (85.5% with ribociclib and 81% with control, HR 0.72, 95% CI 0.62–0.83, *p* < 0.0001). Consistent benefit for node-negative/positive and stage II/III subgroups.

## 7. Future Directions

### 7.1. Limited Adjuvant Endocrine Therapy

Adherence to endocrine therapy for five years is reported to be as low as 58%, likely due to treatment-related side effects and impact on overall quality of life [80]. There is growing interest in ongoing treatment personalization in early stage HR+ breast cancer, including limited duration of adjuvant ET. LA LEAST is a phase II clinical trial (NCT 03917082) that is evaluating a shortened adjuvant ET duration of two years compared to five years in patients aged >50 years with clinically and genomically low-risk HR+ breast cancer as confirmed by Prosigna. The trial has completed accrual, with primary results estimated in 2029. A similar duration of ET is being studied in the French LESS trial using Mammaprint testing to identify low-risk disease (NCT 05297617) [81].

### 7.2. Selective Estrogen Receptor Degraders

Approximately 20% of patients with early stage HR+ breast cancers exhibit innate resistance to ET, resulting in suboptimal benefit with standard ET such as AIs or tamoxifen [82]. In addition, following exposure to ET, up to 40% of cancers develop resistance to these drugs. Of these, roughly 50% are secondary to *ESR1* mutations [83]. *ESR1* mutations are hotspot mutations in the ER, leading to constitutive activation [84]. Selective estrogen receptor degraders (SERDs) offer a promising strategy to overcome this resistance. Unlike SERMS or AIs, SERDs bind directly to the ER and mark it for proteasomal degradation [85].

Fulvestrant, the first SERD developed, has not shown a clear benefit over AIs in early stage HR+ breast cancer. In the GEICAM/2006-10 trial, patients received either five years of adjuvant anastrozole or a combination of fulvestrant and anastrozole for three years, followed by anastrozole alone for two years. No difference in six-year disease-free survival was observed between the groups [86]. Similarly, the ALTERNATE trial evaluated neoadjuvant fulvestrant alone, fulvestrant plus anastrozole, and anastrozole alone in patients with locally advanced HR+ breast cancer. The rates of endocrine-sensitive disease were comparable across groups (23% for fulvestrant, 21% for combination, and 19% for anastrozole alone) [87]. Additional barriers to using fulvestrant include intramuscular administration, which can be inconvenient and may lead to injection-site reactions.

Several oral SERDs (giredestrant, imlunestrant, and elacestrant) are currently under investigation. Both elacestrant and imlunestrant demonstrated a PFS benefit over standard ET in advanced breast cancers with *ESR1* mutations [88,89]. Three ongoing clinical trials are evaluating the efficacy of adjuvant ET with oral SERDs. The ELEGANT trial (NCT 06492616) is assessing the effectiveness of elacestrant in node-positive HR+ early stage breast cancer patients with a high likelihood of recurrence and who have already received two to three years of standard adjuvant ET. The EMBER-4 trial (NCT05514054) is assessing the use of imlunestrant in patients at an increased risk of recurrence who have completed two to five years of standard adjuvant ET [90]. The lidERA trial (NCT04961996) compared giredestrant to standard ET in early HR+ breast cancer. At the San Antonio Breast Cancer Symposium in 2025, giredestrant demonstrated superior three-year invasive DFS compared with standard ET at 92% vs. 90%. Notably, giredestrant seemed to demonstrate increased tolerability, with only 5% of patients discontinuing treatment due to adverse effects compared to 8% with standard ET [91,92].

Beyond SERDs, there are many additional novel ET agents being studied in clinical trials, including proteolysis targeting chimera (PROTACs), novel SERMs, complete ER antagonists (CERAN), and selective estrogen receptor covalent antagonist (SERCA).

### 7.3. PI3K/AKT/mTOR Inhibitors

The PI3K/AKT/mTOR pathway is overactivated in 50% of HR+ breast cancers by means of activating mutations in *PI3K* or *AKT*, or inactivating mutations in *PTEN* [93]. Pathway alterations may be present de novo or acquired by previous treatment.

Chavez-MacGregor et al. investigated whether targeting this pathway could improve outcomes in the adjuvant setting. High-risk HR+ early stage breast cancer patients who received adjuvant or neoadjuvant chemotherapy were randomized to standard ET with or without everolimus, an mTOR inhibitor. Of note, tumours were not biomarker-selected for enrolment in this study. After a median follow-up of 55 months, there were no differences in invasive DFS (74.9% with everolimus vs. 74.4% with ET alone) or OS (88.1% vs. 85.8%) [94]. Notably, only 48% of patients in the everolimus arm completed treatment due to poor tolerability. More than 35% of patients developed grade ≥ 3 adverse events, most commonly stomatitis, cytopenia, hypertriglyceridemia, and hyperglycemia [94].

Capivasertib, an AKT inhibitor, is approved in advanced HR+ breast cancer with alterations in the *PI3K*/*AKT*/*mTOR* pathway following progression after first-line CDK 4/6i and ET [95]. Its role in early stage disease is being explored in the CaptAin trial (NCT 06613516), which is evaluating the addition of capivasertib for two years in patients with detectable circulating tumour DNA after completing standard curative-intent treatment and initiation of adjuvant ET. This phase II clinical trial is ongoing and expected to be completed in 2027.

## 8. Conclusions

Since the earliest observations in the mid 1800s of the benefits of oophorectomy, ET for early stage HR+ breast cancer has advanced significantly. Five years of tamoxifen or AI therapy improves survival and remains the cornerstone of this therapy. Extended or sequential dosing strategies provide additional benefit in selected high-risk patients. In premenopausal women, adding OFS to oral ET enhances disease-free survival, especially in those receiving chemotherapy or with high-risk features (younger age, higher grade). Multiple genomic tests, including Oncotype Dx, MammaPrint, and Prosigna are routinely used to guide adjuvant chemotherapy decisions, and BCI shows utility in determining benefit for extended endocrine therapy. Adjuvant bisphosphonates reduce bone recurrence and mortality in postmenopausal women and are recommended by current guidelines. The CDK 4/6i abemaciclib and ribociclib have demonstrated improved invasive DFS in high-risk early stage HR+ breast cancer patients and are now part of standard adjuvant therapy, including in the lymph node-negative setting for ribociclib. Emerging therapies such as oral SERDs and PI3K/AKT/mTOR inhibitors show promise for improving outcomes in biomarker-selected populations. Ongoing trials will clarify their roles in the adjuvant setting. As adjuvant therapy for early stage HR+ breast cancer continues to evolve, opportunities to personalize treatment to individual risk and tumour biology are expanding, offering the promise of precision oncology.

## 9. Key Findings

- At least five years of adjuvant ET is recommended for women with early stage HR+ breast cancer.
- Five years of an AI or a switch strategy that includes at least two years of an AI is superior to five years of tamoxifen in post-menopausal women.
- Extended-duration endocrine therapy should be considered for patients with high-risk clinical features, and the potential benefit can be predicted using the BCI.
- Adding OFS to ET in premenopausal women improves DFS and OS, particularly among those with high-risk clinical features such as receipt of chemotherapy, age under 35 years, or grade 3 tumours.
- Multi-gene assays can be used as prognostic tools, and some predict the potential benefit of adjuvant chemotherapy.
- Adjuvant bisphosphonates reduce the risk of bone recurrence in postmenopausal women.
- CDK4/6i improve DFS in patients with high-risk, early stage HR+ breast cancer, although OS data remain immature.
- Novel SERDs in the adjuvant setting have begun to demonstrate superior outcomes in disease survival and tolerability in comparison to standard ET.

## Figures and Tables

**Table 3:** Clinical trials for switching treatment.

Clinical Trial	Population	Treatment Regimens	Endpoints	Findings
ABCSG-8/ARNO-95, 2005 [26]	3224 patients	After tamoxifen for 2 years, an additional 3 years of tamoxifen vs. AI	EFS, distant RFS, tolerability	2-year EFS was higher with anastrozole compared to tamoxifen (96% with anastrozole vs. 93% with tamoxifen, HR 0.60, 95% CI 0.44–0.81, *p* = 0.0009).
BIG 1–98, 2011 [18,19,20,21]	8010 patients	Letrozole 2.5 mg PO daily for 5 years vs. tamoxifen 20 mg PO daily for 5 years vs. letrozole for 2 years followed by tamoxifen for 3 years vs. tamoxifen for 2 years followed by letrozole for 3 years	DFS, OS, distant recurrence-free interval, invasive BCFI, breast cancer mortality	No significant differences in the endpoints for either sequence compared to letrozole monotherapy.
IES, 2018 [27]	4724 patients	After 2–3 years of tamoxifen, additional tamoxifen vs. exemestane to complete a total of 5 years	DFS, OS, breast cancer free survival, time to contralateral breast cancer, time to distant recurrence	10-year recurrence rate 22% with exemestane and 26% with tamoxifen (HR 0.81, 95% CI 0.72–0.92, *p* = 0.0006). No significant differences in OS.

## Data Availability

No new data were created or analyzed in this study.

## References

[1] Bray F., Laversanne M., Sung H., Ferlay J., Siegel R.L., Soerjomataram I., Jemal A. (2024). Global cancer statistics 2022: GLOBOCAN estimates of incidence and mortality worldwide for 36 cancers in 185 countries. CA A Cancer J. Clin..

[2] Xiong X., Zheng L.W., Ding Y., Chen Y.F., Cai Y.W., Wang L.P., Huang L., Liu C.C., Shao Z.M., Yu K.D. (2025). Breast cancer: Pathogenesis and treatments. Signal Transduct. Target. Ther..

[3] Waks A.G., Winer E.P. (2019). Breast Cancer Treatment: A Review. JAMA.

[4] Allison K.H., Hammond M.E.H., Dowsett M., McKernin S.E., Carey L.A., Fitzgibbons P.L., Hayes D.F., Lakhani S.R., Chavez-MacGregor M., Perlmutter J. (2020). Estrogen and Progesterone Receptor Testing in Breast Cancer: ASCO/CAP Guideline Update. J. Clin. Oncol..

[5] Beatson G.T. (1896). On the Treatment of Inoperable Cases of Carcinoma of the Mamma: Suggestions for a New Method of Treatment, with Illustrative Cases. Trans. Med. Chir. Soc. Edinb..

[6] Quirke V.M. (2017). Tamoxifen from Failed Contraceptive Pill to Best-Selling Breast Cancer Medicine: A Case-Study in Pharmaceutical Innovation. Front. Pharmacol..

[7] Howell S.J., Johnston S.R., Howell A. (2004). The use of selective estrogen receptor modulators and selective estrogen receptor down-regulators in breast cancer. Best Pract. Res. Clin. Endocrinol. Metab..

[8] Nolvadex Adjuvant Trial Organisation (1988). Controlled trial of tamoxifen as a single adjuvant agent in the management of early breast cancer. Br. J. Cancer.

[9] Fisher B., Dignam J., Bryant J., DeCillis A., Wickerham D.L., Wolmark N., Costantino J., Redmond C., Fisher E.R., Bowman D.M. (1996). Five versus more than five years of tamoxifen therapy for breast cancer patients with negative lymph nodes and estrogen receptor-positive tumors. JNCI J. Natl. Cancer Inst..

[10] Fisher B., Dignam J., Bryant J., Wolmark N. (2001). Five versus more than five years of tamoxifen for lymph node-negative breast cancer: Updated findings from the National Surgical Adjuvant Breast and Bowel Project B-14 randomized trial. JNCI J. Natl. Cancer Inst..

[11] Fisher B., Jeong J.-H., Bryant J., Anderson S., Dignam J., Fisher E.R., Wolmark N. (2004). Treatment of lymph-node-negative, oestrogen-receptor-positive breast cancer: Long-term findings from National Surgical Adjuvant Breast and Bowel Project randomised clinical trials. Lancet.

[12] Fisher B., Redmond C. (1992). Systemic therapy in node-negative patients: Updated findings from NSABP clinical trials. J. Natl. Cancer Inst..

[13] Early Breast Cancer Trialists’ Collaborative Group (2005). Effects of chemotherapy and hormonal therapy for early breast cancer on recurrence and 15-year survival: An overview of the randomised trials. Lancet.

[14] Miller W. (2003). Aromatase inhibitors: Mechanism of action and role in the treatment of breast cancer. Semin. Oncol..

[15] The ATAC (Arimidex, Tamoxifen Alone or in Combination) Trialists’ Group (2002). Anastrozole alone or in combination with tamoxifen versus tamoxifen alone for adjuvant treatment of postmenopausal women with early breast cancer: First results of the ATAC randomised trial. Lancet.

[16] Howell A., Cuzick J., Baum M., Buzdar A., Dowsett M., Forbes J.F., Hoctin-Boes G., Houghton J., Locker G.Y., Tobias J.S. (2005). Results of the ATAC (Arimidex, Tamoxifen, Alone or in Combination) trial after completion of 5 years’ adjuvant treatment for breast cancer. Lancet.

[17] Cuzick J., Sestak I., Baum M., Buzdar A., Howell A., Dowsett M., Forbes J.F. (2010). Effect of anastrozole and tamoxifen as adjuvant treatment for early-stage breast cancer: 10-year analysis of the ATAC trial. Lancet Oncol..

[18] Breast International Group (BIG) (2005). A Comparison of Letrozole and Tamoxifen in Postmenopausal Women with Early Breast Cancer. N. Engl. J. Med..

[19] Coates A.S., Keshaviah A., Thürlimann B., Mouridsen H., Mauriac L., Forbes J.F., Paridaens R., Castiglione-Gertsch M., Gelber R.D., Colleoni M. (2007). Five years of letrozole compared with tamoxifen as initial adjuvant therapy for postmenopausal women with endocrine-responsive early breast cancer: Update of study BIG 1-98. J. Clin. Oncol..

[20] Regan M.M., Neven P., Giobbie-Hurder A., Goldhirsch A., Ejlertsen B., Mauriac L., Forbes J.F., Smith T., Láng I., Wardley A. (2011). Assessment of letrozole and tamoxifen alone and in sequence for postmenopausal women with steroid hormone receptor-positive breast cancer: The BIG 1-98 randomised clinical trial at 8·1 years median follow-up. Lancet Oncol..

[21] Ruhstaller T., Giobbie-Hurder A., Colleoni M., Jensen M.B., Ejlertsen B., de Azambuja E., Neven P., Láng I., Jakobsen E.H., Gladieff L. (2019). Adjuvant Letrozole and Tamoxifen Alone or Sequentially for Postmenopausal Women With Hormone Receptor-Positive Breast Cancer: Long-Term Follow-Up of the BIG 1-98 Trial. J. Clin Oncol..

[22] Smith I., Yardley D., Burris H.A., De Boer R., Amadori D., McIntyre K., Ejlertsen B., Gnant M., Jonat W., Pritchard K.I. (2017). Comparative Efficacy and Safety of Adjuvant Letrozole Versus Anastrozole in Postmenopausal Patients With Hormone Receptor-Positive, Node-Positive Early Breast Cancer: Final Results of the Randomized Phase III Femara Versus Anastrozole Clinical Evaluation (FACE). Trial. J. Clin. Oncol..

[23] Early Breast Cancer Trialists’ Collaborative Group (2015). Aromatase inhibitors versus tamoxifen in early breast cancer: Patient-level meta-analysis of the randomised trials. Lancet.

[24] Chin S.N., Trinkaus M., Simmons C., Flynn C., Dranitsaris G., Bolivar R., Clemons M. (2009). Prevalence and Severity of Urogenital Symptoms in Postmenopausal Women Receiving Endocrine Therapy for Breast Cancer. Clin. Breast Cancer.

[25] Banerjee S., Smith I.E., Folkerd L., Iqbal J., Barker P., Dowsett M. (2005). Comparative effects of anastrozole, tamoxifen alone and in combination on plasma lipids and bone-derived resorption during neoadjuvant therapy in the impact trial. Ann. Oncol..

[26] Jakesz R., Jonat W., Gnant M., Mittlboeck M., Greil R., Tausch C., Hilfrich J., Kwasny W., Menzel C., Samonigg H. (2005). Switching of postmenopausal women with endocrine-responsive early breast cancer to anastrozole after 2 years’ adjuvant tamoxifen: Combined results of ABCSG trial 8 and ARNO 95 trial. Lancet.

[27] Morden J.P., Alvarez I., Bertelli G., Coates A.S., Coleman R., Fallowfield L., Jassem J., Jones S., Kilburn L., Lønning P.E. (2017). Long-Term Follow-Up of the Intergroup Exemestane Study. J. Clin. Oncol..

[28] Davies C., Pan H., Godwin J., Gray R., Arriagada R., Raina V., Abraham M., Medeiros Alencar V.H., Badran A., Bonfill X. (2013). Long-term effects of continuing adjuvant tamoxifen to 10 years versus stopping at 5 years after diagnosis of oestrogen receptor-positive breast cancer: ATLAS, a randomised trial. Lancet.

[29] Gray R.G., Rea D., Handley K., Bowden S.J., Perry P., Earl H.M., Poole C.J., Bates T., Chetiyawardana S., Dewar J.A. (2013). aTTom: Long-term effects of continuing adjuvant tamoxifen to 10 years versus stopping at 5 years in 6953 women with early breast cancer. J. Clin. Oncol..

[30] Goss P.E., Ingle J.N., Pritchard K.I., Robert N.J., Muss H., Gralow J., Gelmon K., Whelan T., Strasser-Weippl K., Rubin S. (2016). Extending aromatase-inhibitor adjuvant therapy to 10 years. N. Engl. J. Med..

[31] Mamounas E.P., Bandos H., Lembersky B.C., Jeong J.-H., Geyer C.E., Rastogi P., Fehrenbacher L., Graham M.L., Chia S.K., Brufsky A.M. (2019). Use of letrozole after aromatase inhibitor-based therapy in postmenopausal breast cancer (NRG Oncology/NSABP B-42): A randomised, double-blind, placebo-controlled, phase 3 trial. Lancet Oncol..

[32] Blok E.J., Kroep J.R., Meershoek-Klein Kranenbarg E., Duijm-de Carpentier M., Putter H., van den Bosch J., Maartense E., van Leeuwen-Stok A.E., Gerrit-Jan L., Nortier J.W.R. (2018). Optimal Duration of Extended Adjuvant Endocrine Therapy for Early Breast Cancer; Results of the IDEAL Trial (BOOG 2006-05). J. Natl. Cancer Inst..

[33] Gnant M., Fitzal F., Rinnerthaler G., Steger G.G., Greil-Ressler S., Balic M., Heck D., Jakesz R., Thaler J., Egle D. (2021). Duration of Adjuvant Aromatase-Inhibitor Therapy in Postmenopausal Breast Cancer. N. Engl. J. Med..

[34] Tjan-Heijnen V.C., Lammers S.W., Geurts S.M., Vriens I.J., Swinkels A.C., Smorenburg C.H., van der Sangen M.J., Kroep J.R., de Graaf H., Honkoop A.H. (2023). Extended adjuvant aromatase inhibition after sequential endocrine therapy in postmenopausal women with breast cancer: Follow-up analysis of the randomised phase. eClinicalMedicine.

[35] Braybrooke J., Bradley R., Hills R.K., Peto R., Kerr A., Pan H., Clarke M., Dodwell D., McGale P., Taylor C. (2025). Extending the duration of endocrine treatment for early breast cancer: Patient-level meta-analysis of 12 randomised trials of aromatase inhibitors in 22031 postmenopausal women already treated with at least 5 years of endocrine therapy. Lancet.

[36] Sinha S., Kaseta J., Santner S., Demers L., Bremmer W., Santen R. (1998). Effect of CGS 20267 on ovarian aromatase and gonadotropin levels in the rat. Breast Cancer Res. Treat..

[37] Pagani O., Regan M.M., Walley B.A., Fleming G.F., Colleoni M., Láng I., Gomez H.L., Tondini C., Burstein H.J., Perez E.A. (2014). Adjuvant Exemestane with Ovarian Suppression in Premenopausal Breast Cancer. N. Engl. J. Med..

[38] Francis P.A., Pagani O., Fleming G.F., Walley B.A., Colleoni M., Láng I., Gómez H.L., Tondini C., Ciruelos E., Burstein H.J. (2018). Tailoring adjuvant endocrine therapy for premenopausal breast cancer. N. Engl. J. Med..

[39] Pagani O., Walley B.A., Fleming G.F., Colleoni M., Láng I., Gomez H.L., Brezden-Masley C., Puhalla S., Linderholm B., Nucifora P. (2022). Adjuvant Exemestane With Ovarian Suppression in Premenopausal Breast Cancer: Long-Term Follow-Up of the Combined TEXT and SOFT Trials. J. Clin. Oncol..

[40] Francis P.A., Fleming G.F., Pagani O., Walley B., Loi S., Colleoni M., Regan M.M. (2025). 15-year outcomes for women with premenopausal hormone receptor-positive early breast cancer (BC) in the SOFT and TEXT trials assessing benefits from adjuvant exemestane (E) + ovarian function suppression (OFS) or tamoxifen (T)+OFS. J. Clin. Oncol..

[41] Paik S., Tang G., Shak S., Kim C., Baker J., Kim W., Cronin M., Baehner F.L., Watson D., Bryant J. (2006). Gene expression and benefit of chemotherapy in women with node-negative, estrogen receptor-positive breast cancer. J. Clin. Oncol..

[42] Sparano J.A., Gray R.J., Makower D.F., Pritchard K.I., Albain K.S., Hayes D.F., Geyer C.E., Dees E.C., Goetz M.P., Olson J.A. (2018). Adjuvant Chemotherapy Guided by a 21-Gene Expression Assay in Breast Cancer. N. Engl. J. Med..

[43] Kalinsky K., Barlow W.E., Gralow J.R., Meric-Bernstam F., Albain K.S., Hayes D.F., Lin N.U., Perez E.A., Goldstein L.J., Chia S.K.L. (2021). 21-Gene Assay to Inform Chemotherapy Benefit in Node-Positive Breast Cancer. N. Engl. J. Med..

[44] Swain S.M., Tang G., Puhalla S.L., Ganz P.A., Henry N.L., Cecchini R.S., Reid S.A., Rastogi P., Geyer C.E., White J.R. (2024). A phase III trial evaluating addition of adjuvant chemotherapy to ovarian function suppression + endocrine therapy in premenopausal women with pN0-1, HR+/HER2- breast cancer (BC) and oncotype recurrence score (RS) ≤25 (OFSET): NRG-BR009. J. Clin. Oncol..

[45] Cardoso F., van’t Veer L.J., Bogaerts J., Slaets L., Viale G., Delaloge S., Pierga J.Y., Brain E., Causeret S., Delorenzi M. (2016). 70-Gene Signature as an Aid to Treatment Decisions in Early-Stage Breast Cancer. N. Engl. J. Med..

[46] Piccart M., Veer L.J.v.’., Poncet C., Cardozo J.M.N.L., Delaloge S., Pierga J.-Y., Vuylsteke P., Brain E., Vrijaldenhoven S., A Neijenhuis P. (2021). 70-gene signature as an aid for treatment decisions in early breast cancer: Updated results of the phase 3 randomised MINDACT trial with an exploratory analysis by age. Lancet Oncol..

[47] Nielsen T., Wallden B., Schaper C., Ferree S., Liu S., Gao D., Barry G., Dowidar N., Maysuria M., Storhoff J. (2014). Analytical validation of the PAM50-based Prosigna Breast Cancer Prognostic Gene Signature Assay and nCounter Analysis System using formalin-fixed paraffin-embedded breast tumor specimens. BMC Cancer.

[48] Wallden B., Storhoff J., Nielsen T., Dowidar N., Schaper C., Ferree S., Liu S., Leung S., Geiss G., Snider J. (2015). Development and verification of the PAM50-based Prosigna breast cancer gene signature assay. BMC Med. Genom..

[49] Sestak I., Buus R., Cuzick J., Dubsky P., Kronenwett R., Denkert C., Ferree S., Sgroi D., Schnabel C., Baehner F.L. (2018). Comparison of the performance of 6 prognostic signatures for estrogen receptor–positive breast cancer: A secondary analysis of a randomized clinical trial. JAMA Oncol..

[50] Jensen M.-B., Lænkholm A.-V., Balslev E., Buckingham W., Ferree S., Glavicic V., Jensen J.D., Knoop A.S., Mouridsen H.T., Nielsen D. (2020). The Prosigna 50-gene profile and responsiveness to adjuvant anthracycline-based chemotherapy in high-risk breast cancer patients. NPJ Breast Cancer.

[51] Sgroi D.C., Carney E., Zarrella E., Steffel L., Binns S.N., Finkelstein D.M., Szymonifka J., Bhan A.K., Shepherd L.E., Zhang Y. (2013). Prediction of late disease recurrence and extended adjuvant letrozole benefit by the HOXB13/IL17BR biomarker. J. Natl. Cancer Inst..

[52] Bartlett J., Sgroi D., Treuner K., Zhang Y., Ahmed I., Piper T., Salunga R., Brachtel E., Pirrie S., Schnabel C. (2019). Breast Cancer Index and prediction of benefit from extended endocrine therapy in breast cancer patients treated in the Adjuvant Tamoxifen—To Offer More?(aTTom) trial. Ann. Oncol..

[53] Bartlett J.M.S., Sgroi D.C., Treuner K., Zhang Y., Piper T., Salunga R.C., Schnabel C.A., Hayes D.F., Dowsett M., Regan M.M. (2022). Breast Cancer Index Is a Predictive Biomarker of Treatment Benefit and Outcome from Extended Tamoxifen Therapy: Final Analysis of the Trans-aTTom Study. Clin. Cancer Res..

[54] Buus R., Sestak I., Kronenwett R., Ferree S., Schnabel C.A., Baehner F.L., Mallon E.A., Cuzick J., Dowsett M. (2021). Molecular Drivers of Oncotype DX, Prosigna, EndoPredict, and the Breast Cancer Index: A TransATAC Study. J. Clin. Oncol..

[55] Dabbs D.J., Brufsky A., Jankowitz R.C., Puhalla S., Lee A., Oesterreich S., Lembersky B.C., Bhargava R. (2014). Comparison of test results and clinical outcomes of patients assessed with both MammaPrint and Oncotype DX with pathologic variables: An independent study. J. Clin. Oncol..

[56] Maroun R., Saleh R., Asselah J., Omeroglu A., Gulbeyaz A., Meterissian S.H., Bouganim N. (2015). A head-to-head comparison of Mammaprint and Oncotype Dx: A McGill University Health Center Experience. J. Clin. Oncol..

[57] Coleman R.E. (2006). Clinical features of metastatic bone disease and risk of skeletal morbidity. Clin Cancer Res.

[58] Mundy G. (2002). Metastasis to bone: Causes, consequences and therapeutic opportunities. Nat. Rev. Cancer.

[59] Coleman R.E., Croucher P.I., Padhani A.R., Clézardin P., Chow E., Fallon M., Guise T., Colosia A., Cleeland C., Costa L. (2020). Bone metastases. Nat. Rev. Dis. Primers.

[60] Paterson A.H., Anderson S.J., Lembersky B.C., Fehrenbacher L., I Falkson C., King K.M., Weir L.M., Brufsky A.M., Dakhil S., Lad T. (2012). Oral clodronate for adjuvant treatment of operable breast cancer (National Surgical Adjuvant Breast and Bowel Project protocol B-34): A multicentre, placebo-controlled, randomised trial. Lancet Oncol..

[61] Coleman R., Cameron D., Dodwell D., Bell R., Wilson C., Rathbone E., Keane M., Gil M., Burkinshaw R., Grieve R. (2014). Adjuvant zoledronic acid in patients with early breast cancer: Final efficacy analysis of the AZURE (BIG 01/04) randomised open-label phase 3 trial. Lancet Oncol..

[62] Gnant M., Mlineritsch B., Stoeger H., Luschin-Ebengreuth G., Knauer M., Moik M., Jakesz R., Seifert M., Taucher S., Bjelic-Radisic V. (2015). Zoledronic acid combined with adjuvant endocrine therapy of tamoxifen versus anastrozol plus ovarian function suppression in premenopausal early breast cancer: Final analysis of the Austrian Breast and Colorectal Cancer Study Group Trial 12. Ann. Oncol..

[63] Early Breast Cancer Trialists’ Collaborative Group (2015). Adjuvant bisphosphonate treatment in early breast cancer: Meta-analyses of individual patient data from randomised trials. Lancet.

[64] Eisen A., Somerfield M.R., Accordino M.K., Blanchette P.S., Clemons M.J., Dhesy-Thind S., Dillmon M.S., D’ORonzo S., Fletcher G.G., Frank E.S. (2022). Use of Adjuvant Bisphosphonates and Other Bone-Modifying Agents in Breast Cancer: ASCO-OH (CCO) Guideline Update. J. Clin. Oncol..

[65] Gnant M., Pfeiler G., Dubsky P.C., Hubalek M., Greil R., Jakesz R., Wette V., Balic M., Haslbauer F., Melbinger-Zeinitzer E. (2015). Adjuvant denosumab in breast cancer (ABCSG-18): A multicentre, randomised, double-blind, placebo-controlled trial. Lancet.

[66] Gnant M., Frantal S., Pfeiler G., Steger G.G., Egle D., Greil R., Fitzal F., Wette V., Balic M., Haslbauer F. (2022). Long-Term Outcomes of Adjuvant Denosumab in Breast Cancer. NEJM Evid..

[67] Coleman R., Finkelstein D.M., Barrios C., Martin M., Iwata H., Hegg R., Glaspy J., Periañez A.M., Tonkin K., Deleu I. (2020). Adjuvant denosumab in early breast cancer (D-CARE): An international, multicentre, randomised, controlled, phase 3 trial. Lancet Oncol..

[68] Friedl T.W.P., Fehm T., Müller V., Lichtenegger W., Blohmer J., Lorenz R., Forstbauer H., Fink V., Bekes I., Janni W. (2021). Prognosis of Patients With Early Breast Cancer Receiving 5 Years vs 2 Years of Adjuvant Bisphosphonate Treatment: A Phase 3 Randomized Clinical Trial. JAMA Oncol..

[69] Scott S.C., Lee S.S., Abraham J. (2017). Mechanisms of therapeutic CDK4/6 inhibition in breast cancer. Semin. Oncol..

[70] Mayer E.L., Dueck A.C., Martin M., Rubovszky G., Burstein H.J., Bellet-Ezquerra M., Miller K.D., Zdenkowski N., Winer E.P., Pfeiler G. (2021). Palbociclib with adjuvant endocrine therapy in early breast cancer (PALLAS): Interim analysis of a multicentre, open-label, randomised, phase 3 study. Lancet Oncol..

[71] Gnant M., Dueck A.C., Frantal S., Martin M., Burstein H.J., Greil R., Fox P., Wolff A.C., Chan A., Winer E.P. (2022). Adjuvant Palbociclib for Early Breast Cancer: The PALLAS Trial Results (ABCSG-42/AFT-05/BIG-14-03). J. Clin. Oncol..

[72] Loibl S., Marmé F., Martin M., Untch M., Bonnefoi H., Kim S.-B., Bear H., McCarthy N., Olivé M.M., Gelmon K. (2021). Palbociclib for Residual High-Risk Invasive HR-Positive and HER2-Negative Early Breast Cancer—The Penelope-B Trial. J. Clin. Oncol..

[73] Johnston S.R.D., Toi M., O’SHaughnessy J., Rastogi P., Campone M., Neven P., Huang C.-S., Huober J., Jaliffe G.G., Cicin I. (2023). Abemaciclib plus endocrine therapy for hormone receptor-positive, HER2-negative, node-positive, high-risk early breast cancer (monarchE): Results from a preplanned interim analysis of a randomised, open-label, phase 3 trial. Lancet Oncol..

[74] Rastogi P., O’SHaughnessy J., Martin M., Boyle F., Cortes J., Rugo H.S., Goetz M.P., Hamilton E.P., Huang C.-S., Senkus E. (2024). Adjuvant Abemaciclib Plus Endocrine Therapy for Hormone Receptor-Positive, Human Epidermal Growth Factor Receptor 2-Negative, High-Risk Early Breast Cancer: Results From a Preplanned monarchE Overall Survival Interim Analysis, Including 5-Year Efficacy Outcomes. J. Clin. Oncol..

[75] Johnston S., Martin M., O’Shaughnessy J., Hegg R., Tolaney S.M., Guarneri V., Huober J., DeCensi A., Sledge G.W., Rugo H.S. (2025). Overall survival with abemaciclib in early breast cancer. Ann. Oncol..

[76] Johnston S.R.D., Harbeck N., Hegg R., Toi M., Martin M., Shao Z.M., Zhang Q.Y., Rodriguez J.L.M., Campone M., Hamilton E. (2020). Abemaciclib Combined With Endocrine Therapy for the Adjuvant Treatment of HR+, HER2-, Node-Positive, High-Risk, Early Breast Cancer (monarchE). J. Clin. Oncol..

[77] Slamon D., Lipatov O., Nowecki Z., McAndrew N., Kukielka-Budny B., Stroyakovskiy D., Yardley D.A., Huang C.-S., Fasching P.A., Crown J. (2024). Ribociclib plus Endocrine Therapy in Early Breast Cancer. N. Engl. J. Med..

[78] Hortobagyi G., Lacko A., Sohn J., Cruz F., Borrego M.R., Manikhas A., Park Y.H., Stroyakovskiy D., Yardley D., Huang C.-S. (2025). A phase III trial of adjuvant ribociclib plus endocrine therapy versus endocrine therapy alone in patients with HR-positive/HER2-negative early breast cancer: Final invasive disease-free survival results from the NATALEE trial. Ann. Oncol..

[79] Crown J., Stroyakovskii D., Yardley D., Huang C.-S., Fasching P., Bardia A., Chia S., Im S.-A., Martin M., Xu B. (2025). Adjuvant ribociclib plus nonsteroidal aromatase inhibitor therapy in patients with HR-positive/HER2-negative early breast cancer: 5-year follow-up of NATALEE efficacy outcomes and updated overall survival. ESMO Open.

[80] Assad-Suzuki D., Laperche-Santos D., Resende H., Moura F.C., Oliveira S.C.S., Shimada A.K., Arakelian R., Galvão A.L.Z., de Souza B.S.W., Custódio A.G.C. (2025). Adherence to Adjuvant Endocrine Therapy in Patients With Nonmetastatic Estrogen Receptor–Positive Breast Cancer: A Comprehensive Brazilian Real-World Data Study. JCO Glob. Oncol..

[81] Deluche E., Michiels S., Fric D., Perrin C., Bailleux C., Bachelot T., Yun P.K.K., De Rauglaudre G., Mouret-Reynier M.-A., Le Scodan R. (2023). LESS: Single-arm study to de-escalate adjuvant endocrine therapy duration in post-menopausal women with HR+ HER2- early-stage breast cancer at very low risk of metastasis. J. Clin. Oncol..

[82] Anurag M., Ellis M.J., Haricharan S. (2018). DNA damage repair defects as a new class of endocrine treatment resistance driver. Oncotarget.

[83] Brett J.O., Spring L.M., Bardia A., Wander S.A. (2021). ESR1 mutation as an emerging clinical biomarker in metastatic hormone receptor-positive breast cancer. Breast Cancer Res..

[84] Toy W., Shen Y., Won H., Green B., Sakr R.A., Will M., Li Z., Gala K., Fanning S., King T.A. (2013). ESR1 ligand-binding domain mutations in hormone-resistant breast cancer. Nat. Genet..

[85] Patel H.K., Bihani T. (2018). Selective estrogen receptor modulators (SERMs) and selective estrogen receptor degraders (SERDs) in cancer treatment. Pharmacol. Ther..

[86] Ruíz-Borrego M., Guerrero-Zotano A., Bermejo B., Ramos M., Cruz J., Baena-Cañada J.M., Cirauqui B., Rodríguez-Lescure Á., Alba E., Martínez-Jáñez N. (2019). Phase III evaluating the addition of fulvestrant (F) to anastrozole (A) as adjuvant therapy in postmenopausal women with hormone receptor-positive HER2-negative (HR+/HER2−) early breast cancer (EBC): Results from the GEICAM/2006–10 study. Breast Cancer Res. Treat..

[87] Ma C.X., Suman V.J., Sanati S., Vij K., Anurag M., Leitch A.M., Unzeitig G.W., Hoog J., Fernandez-Martinez A., Fan C. (2024). Endocrine-Sensitive Disease Rate in Postmenopausal Patients With Estrogen Receptor–Rich/ERBB2-Negative Breast Cancer Receiving Neoadjuvant Anastrozole, Fulvestrant, or Their Combination: A Phase 3 Randomized Clinical Trial. JAMA Oncol..

[88] Bidard F.-C., Kaklamani V.G., Neven P., Streich G., Montero A.J., Forget F., Mouret-Reynier M.-A., Sohn J.H., Taylor D., Harnden K.K. (2022). Elacestrant (oral selective estrogen receptor degrader) Versus Standard Endocrine Therapy for Estrogen Receptor–Positive, Human Epidermal Growth Factor Receptor 2–Negative Advanced Breast Cancer: Results From the Randomized Phase III EMERALD Trial. J. Clin. Oncol..

[89] Jhaveri K.L., Neven P., Casalnuovo M.L., Kim S.-B., Tokunaga E., Aftimos P., Bachelot T., Hamilton E.P., Loi S., Oliveira M. (2025). Imlunestrant with or without Abemaciclib in Advanced Breast Cancer. N. Engl. J. Med..

[90] Jhaveri K., O’sHaughnessy J., Andre F., Goetz M.P., Harbeck N., Martín M., Bidard F.-C., Thomas Z.M., Young S., Ismail-Khan R. (2023). Abstract OT1-01-02: EMBER-4: A phase 3 adjuvant trial of imlunestrant vs standard endocrine therapy (ET) in patients with ER+, HER2- early breast cancer (EBC) with an increased risk of recurrence who have previously received 2 to 5 years of adjuvant ET. Cancer Res..

[91] Geyer C., Bardia A., Harbeck N., Rimawi M.F., Hurvitz S.A., Martin M., Loi S., Saji S., Jung K.H., Werutsky G. (2023). lidERA Breast Cancer (BC): Phase III adjuvant study of giredestrant vs. physician’s choice of endocrine therapy (PCET) in patients (pts) with estrogen receptor-positive, HER2-negative early BC (ER+, HER2– eBC). J. Clin. Oncol..

[92] Bardia A., Schmid P., Martín M., Hurvitz S., Jung K., Rimawi M., Saji S., Werutsky G., Harbeck N., Loi S. (2025). Giredestrant vs standard-of-care endocrine therapy as adjuvant treatment for patients with estrogen receptor-positive, HER2-negative early breast cancer: Results from the global Phase III lidERA Breast Cancer trial. San Antonio Breast Cancer Symp..

[93] Li H., Prever L., Hirsch E., Gulluni F. (2021). Targeting PI3K/AKT/mTOR Signaling Pathway in Breast Cancer. Cancers.

[94] Chavez-MacGregor M., Miao J., Pusztai L., Goetz M.P., Rastogi P., Ganz P.A., Mamounas E.P., Paik S., Bandos H., Razaq W. (2024). Phase III Randomized, Placebo-Controlled Trial of Endocrine Therapy ± 1 Year of Everolimus in Patients With High-Risk, Hormone Receptor-Positive, Early-Stage Breast Cancer. J. Clin. Oncol..

[95] Turner N.C., Oliveira M., Howell S.J., Dalenc F., Cortes J., Moreno H.L.G., Hu X., Jhaveri K., Krivorotko P., Loibl S. (2023). Capivasertib in Hormone Receptor–Positive Advanced Breast Cancer. N. Engl. J. Med..

